# Supplementary material for: A histone demethylase links the loss of plasticity to nongenetic inheritance and morphological change
Source: Nat Commun. 2023 Dec 19;14:8439. doi: 10.1038/s41467-023-44306-8 (PMC10730525; doi:10.1038/s41467-023-44306-8)
Supplement: Supplementary file 1 — Supplementary Information file [file 41467_2023_44306_MOESM1_ESM.pdf]

Supplementary Information for

A histone demethylase links the loss of plasticity to nongenetic inheritance and morphological change

Nicholas A. Levis[1,*] and Erik J. Ragsdale[1,*]

---

[1]Department of Biology, Indiana University, Bloomington, IN 47405, USA

*e-mail: nicholasalevis@gmail.com, ragsdale@indiana.edu

Supplementary Table 1. Summary of selection signatures for genes and orthogroups for each species and putative assimilation event.

Species	Episodic, diversifying selection	Selection intensity
P. bucculentus	1298 (22; 17)	778 (16; 6)
P. elegans	1548 (33; 19)	835 (17; 12)
D. magnus	1417 (32; 21)	1779 (35; 18)
L. texanum	1418 (22; 13)	1531 (38; 20)
P. bucculentus + *P. elegans*	551 (13; 7)	231 (7; 4)
D. magnus + *L. texanum*	335 (5; 3)	506 (17; 5)

Parentheses indicate the number of Eu-associated and St-associated genes or orthogroups. The number of significant polyphenism or morph-associated genes and orthogroups was not significantly different than expected by chance (one-sided goodness-of-fit test) in all cases except for *D. magnus* + *L. texanum* selection intensity. There, Eu-associated orthogroups were over-represented ($\chi^2 = 8.67$; $P = 0.013$).

Supplementary Table 2. crRNA and primer sequences for *Pristionchus fissidentatus* CRISPR/Cas9 edits.

Sequence type	*eud-1*	*seud-1*
Target (with PAM site)	ATGTGCAGTCCCTCAAGAGCTGG	TTTCTTCGAATTGTTCATGCAGG
Forward primer	GGGCGTCCAGTCTTTGTTAT	GGAGCGGCAAAGTACATCTAT
Reverse primer	CCCGTAACTCCAATTCTGATAGG	ACACTCCACTCTTCTGGTTTG

Supplementary Table 3. crRNA, repair template, and primer sequences for *P. pacificus spr-5* CRISPR/Cas9 knock-in.

Sequence type	Sequence
Target (with PAM site)	GCCAAACAATTAAAACGATTCGG
Repair template	ATTCTAACTGCAG
Forward primer	TGTTGCAATGGGAATATATGGA
Reverse primer	GACTTCCCGATAATACTCCGTAATAA

Supplementary Table 4. Description of landmarks used for geometric morphometrics.

Number	Morphological description	Landmark type
1	Anteriormost point of cheilostom on dorsal side	Homologous
2	Posteriormost point of cheilostom on dorsal side	Homologous
3	Anteriormost point of gymnostom on dorsal side	Homologous
4	Posteriormost point of gymnostom on dorsal side	Homologous
5	Anteriormost point of prostegostom on dorsal side	Homologous
6	Extreme point of concave part of dorsal surface of dorsal tooth	Sliding
7	Apex of convex part of dorsal surface of dorsal tooth	Sliding
8	Tip of dorsal tooth	Homologous
9	Dorsal pharyngeal gland orifice	Homologous
10	Bottom of edge of dorsal tooth hook	Sliding
11	Apex of convex part of ventral surface of dorsal tooth	Homologous
12	Junction between dorsal tooth and right subventral tooth/ridge	Homologous
13	Dorsal base of hook of right subventral tooth/denticle	Sliding
14	Tip of hook of right subventral tooth/denticle	Homologous
15	Ventral base of hook of right subventral tooth/denticle	Sliding
16	Extreme point of concave part of right subventral tooth/ridge	Sliding
17	Ventral base of right subventral tooth/ridge	Homologous
18	Posteriormost point of gymnostom on ventral side	Homologous
19	Anteriormost point of prostegostom on ventral side	Homologous
20	Anteriormost point of gymnostom on ventral side	Homologous
21	Posteriormost point of cheilostom on ventral side	Homologous
22	Anteriormost point of cheilostom on ventral side	Homologous

Landmarks follow Sieriebriennikov *et al*. (2017)[81], except for 10 and 16, which are original to this study.

RS5133 TGAAGGCACGAGATTCACAAATGCCTATTCTGCTGATAGTATGTGCAGTCCCTCAAGAGCTGGATTC
Pfi-eud-1(iub27) TGAAGGCACGAAGG--CAGCTGGATTC
***********   **********

RS5133 CACGGCGAATTCGATGTTTTCTTCGAATTGTTCATGCAGGGAAAGGTTCGATTTATTGATTTTTTTT
Pfi-seud-1(iub22) CACGGCGAATTCGATGTTTTCTTCGAATT------GCAGGGAAAGGTTCGATTTATTGATTTTTTTT
*****************************   ********************************

PS312 GACTGCGGCCAAACAATTAAAACGAT-------------TCGGATTCGATGTTACCATCCTCGAAAT
Ppa-spr-5(iub33) GACTGCGGCCAAACAATTAAAACGATTC**TAA**CTGCAGATTCGGATTCGATGTTACCATCCTCGAAAT
**************************   ****************************

RS5200B TGGAATGACTGCGGCCAAACAATTAAAACGATTCGGATTCGATGTTACCATCCTCGAAATGAGAGTG
Ppa-spr-5(iub44) TGGAATGACTGCGGCCAAACAATTAAAA-----CGGATTCGATGTTACCATCCTCGAAATGAGAGTG
****************************   **********************************

Supplementary Fig. 1. Mutant alleles generated by CRISPR/Cas9. Alignments are of sequences containing mutations in *Pristionchus fissidentatus* (*Pfi*) and *P. pacificus* (*Ppa*). Boldface font indicates inserted stop codon.

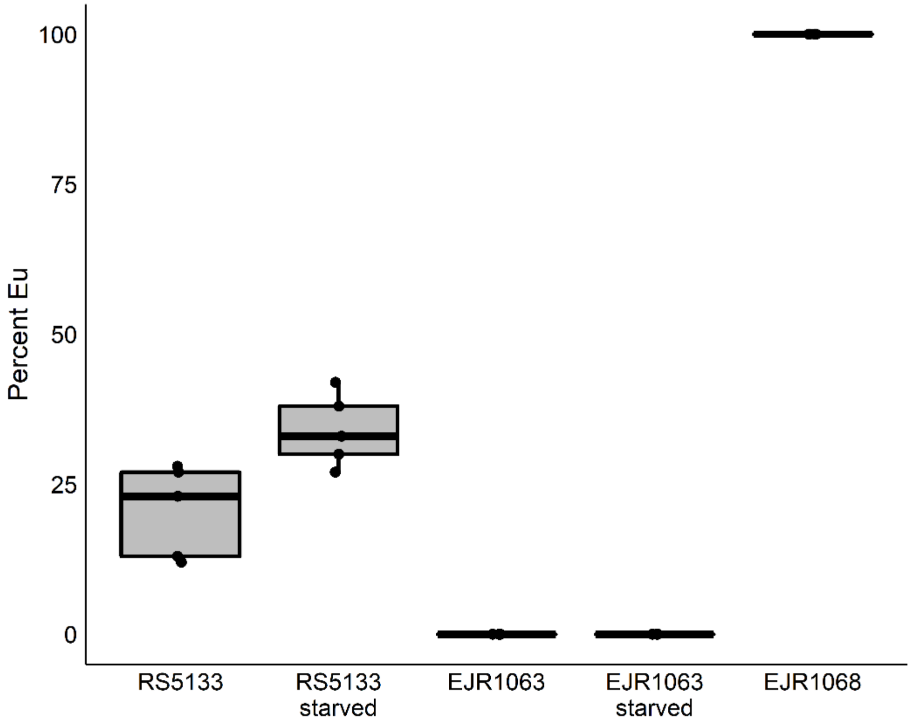

Supplementary Fig. 2. Confirmation of polyphenism switch-gene function in *Pristionchus fissidentatus*. Whereas wild-type *P. fissidentatus* (strain RS5133) increase the proportion of Eu individuals under starvation conditions, knockout of the gene *eud-1* (allele *iub27*, strain EJR1063) causes loss of the Eu morph even when starved. Knockout of *seud-1* (allele *iub22*, strain EJR1068) causes fixation of the Eu morph even under non-starvation conditions. Five replicate plates (solid points) of 60 individuals were assayed for each group. The lower and upper hinges of the box plots correspond to the first and third quartiles, the whiskers extend to the largest value no further than 1.5x the inter-quartile range from the hinges, and the centre corresponds to the median. Source data are provided as a Source Data file.

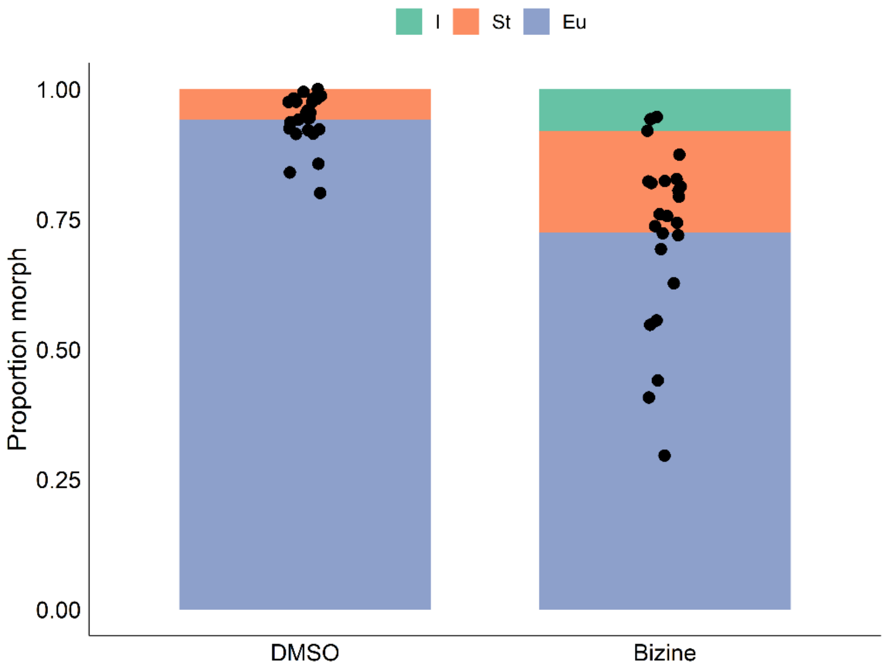

Supplementary Fig. 3. Treatment with SPR-5 inhibitor Bizine phenocopies *spr-5* genetic ablation in *P. pacificus* (PS312). Two-tailed logistic regression revealed that compared to controls treated with DMSO, plates treated with Bizine showed a decrease in the frequency of Eu individuals and a concomitant increase in St and intermediate morphs (estimate = 1.32, $Z = 3.68$ $P = 2.33 \times 10^{-4}$). In addition, the absolute number of intermediates (I) produced in the Bizine treatment was significantly higher than the controls according to Fisher's exact test (96 in Bizine treatment, zero in control; $P = 2.20 \times 10^{-16}$). Bars are the aggregate proportions from eight generations where each generation, for each treatment, consisted of three biological replicates of 50 individuals ($n = 150$ per generation, 1200 total individuals). Solid points represent the percent Eu per replicate. Source data are provided as a Source Data file.
