# Supplementary material for: A histone demethylase links the loss of plasticity to nongenetic inheritance and morphological change
Source: Nat Commun. 2023 Dec 19;14:8439. doi: 10.1038/s41467-023-44306-8 (PMC10730525; doi:10.1038/s41467-023-44306-8)
Supplement: Supplementary file 3 — Reporting Summary [file 41467_2023_44306_MOESM3_ESM.pdf]

nature portfolio

Corresponding author(s): Nicholas A. Levis & Erik J. Ragsdale

Last updated by author(s): Nov 21, 2023

Reporting Summary

Nature Portfolio wishes to improve the reproducibility of the work that we publish. This form provides structure for consistency and transparency in reporting. For further information on Nature Portfolio policies, see our Editorial Policies and the Editorial Policy Checklist.

Statistics

For all statistical analyses, confirm that the following items are present in the figure legend, table legend, main text, or Methods section.

n/a	Confirmed	
☐	☒	The exact sample size (n) for each experimental group/condition, given as a discrete number and unit of measurement
☐	☒	A statement on whether measurements were taken from distinct samples or whether the same sample was measured repeatedly
☐	☒	The statistical test(s) used AND whether they are one- or two-sided<br>*Only common tests should be described solely by name; describe more complex techniques in the Methods section.*
☐	☒	A description of all covariates tested
☐	☒	A description of any assumptions or corrections, such as tests of normality and adjustment for multiple comparisons
☐	☒	A full description of the statistical parameters including central tendency (e.g. means) or other basic estimates (e.g. regression coefficient) AND variation (e.g. standard deviation) or associated estimates of uncertainty (e.g. confidence intervals)
☐	☒	For null hypothesis testing, the test statistic (e.g. F, t, r) with confidence intervals, effect sizes, degrees of freedom and P value noted<br>*Give P values as exact values whenever suitable.*
☒	☐	For Bayesian analysis, information on the choice of priors and Markov chain Monte Carlo settings
☒	☐	For hierarchical and complex designs, identification of the appropriate level for tests and full reporting of outcomes
☒	☐	Estimates of effect sizes (e.g. Cohen's d, Pearson's r), indicating how they were calculated

Our web collection on statistics for biologists contains articles on many of the points above.

Software and code

Policy information about availability of computer code

Data collection	Transcriptomes were accessed from http://pristionchus.org/download/Roedelsperger_et_al_2018_pristionchus_transcriptome_assemblies.tgz. Genomes were accessed from http://pristionchus.org/download/diplogastrid_annotation_ppcac_v1.tgz, http://pristionchus.org/download/El_Paco_genome.tgz, and from NCBI (PRJNA655932). Sequences were processed using standard tools such as MAFFT (v. 7.471), MACSE (v. 2.03), Divvier (v. 1.01), RAxML (v. 8.2.11), and OrthoFinder (v. 2.5.1) to align, filter, and group genes for tests of diversifying or weakened selection. Images for geometric morphometrics were acquired using Zeiss Zen (2012) software and were processed using Fiji.
Data analysis	Tests of selection were done using HyPhy (v. 2.5.21), statistical analyses were performed using R (v. 4.0), and ad hoc tests of diversifying selection used PAMLX (v. 1.3.1). Protein modeling used the Phyre2 online tool. Scripts for statistical analysis can be accessed via Dryad Digital Repository (DOI: 10.5061/dryad.98sf7m0nm).

For manuscripts utilizing custom algorithms or software that are central to the research but not yet described in published literature, software must be made available to editors and reviewers. We strongly encourage code deposition in a community repository (e.g. GitHub). See the Nature Portfolio guidelines for submitting code & software for further information.

Data

Policy information about availability of data

All manuscripts must include a data availability statement. This statement should provide the following information, where applicable:

- Accession codes, unique identifiers, or web links for publicly available datasets
- A description of any restrictions on data availability
- For clinical datasets or third party data, please ensure that the statement adheres to our policy

Transcriptomes were accessed from pristionchus.org (http://pristionchus.org/download/Roedelsperger_et_al_2018_pristionchus_transcriptome_assemblies.tgz). Genomes were accessed from pristionchus.org (http://pristionchus.org/download/diplogastrid_annotation_ppcac_v1.tgz, http://pristionchus.org/download/El_Paco_genome.tgz) and from NCBI (https://www.ncbi.nlm.nih.gov/nuccore?term=PRJNA655932). Newly generated RNA sequencing reads have been deposited in the NCBI Sequence Read Archive (PRJNA919017). Lists of polyphenism-associated genes, genes under selection, phenotypic data, and code used to analyze data are available in the Dryad Digital Repository (DOI: 10.5061/dryad.98sf7m0nm). All other data needed to evaluate the conclusions in the paper are present in the paper, the Supplementary Information, or the Source Data file.

Human research participants

Policy information about studies involving human research participants and Sex and Gender in Research.

Reporting on sex and gender	N/A
Population characteristics	N/A
Recruitment	N/A
Ethics oversight	N/A

Note that full information on the approval of the study protocol must also be provided in the manuscript.

Field-specific reporting

Please select the one below that is the best fit for your research. If you are not sure, read the appropriate sections before making your selection.

☐ Life sciences ☐ Behavioural & social sciences ☒ Ecological, evolutionary & environmental sciences

For a reference copy of the document with all sections, see nature.com/documents/nr-reporting-summary-flat.pdf

Ecological, evolutionary & environmental sciences study design

All studies must disclose on these points even when the disclosure is negative.

Study description	This study identified genes experiencing evolutionary change associated with independent losses of plasticity, using published genomes and transcriptomes. Upon identifying candidates, the list was narrowed by comparing it with genes that are associated with polyphenism in the genus Pristionchus. These polyphenism genes were identified via RNA-seq of alternative morphs, specifically by comparing the transcriptomes of morph-constitutive lines engineered by DNA-editing in multiple Pristionchus species. We performed functional tests (using CRISPR/Cas9 modifications) to evaluate the role of one key target, spr-5. The phenotypic effects of this gene on a polyphenic species and a monomorphic line were evaluated qualitatively as the proportions of alternative adult morphs and quantitatively using geometric morphometrics. Morphometric data were collected on adults of varying genotypes where genotype and qualitative morph were used as factors for analysis. We also evaluated the gene's effects on plasticity by quantifying morph induction under different rearing conditions for mutant and wild-type genotypes (i.e., using a factorial design). These rearing conditions included liquid media (M9 buffer), solid media (nematode growth medium agar), ad libitum feeding, and starvation. We then performed a controlled cross and observed immediate phenotypic changes and changes after 10 generations of selecting for or against a specific (eurystomatous) morphology. To score phenotypes, individuals were nested into replicate plates or vials and 30-60 individuals were phenotyped per replicate, with the number of replicates ranging from 3-5 for a given treatment. In the artificial selection portion of the study, all individuals on a plate were phenotyped if there were fewer than 50 adults and this reached as few as seven individuals.
Research sample	We used Pristionchus fissidentatus to help identify a core set of polyphenism associated genes because it had an early divergence from Pristionchus pacificus. We validated candidate assimilation associated genes in Prisionchus pacificus because it is the standard model for this genus/family of nematodes and is the most well-studied.
Sampling strategy	Adults were haphazardly visually assessed for morphology. For geometric morphometrics, adults were haphazardly picked onto slides for imaging. We followed sample size conventions of previous studies (e.g., Ragsdale et al., 2013 Cell 155, 922–933; Werner et al., 2017 Scientific Reports 7, 7207; Bui et al., 2018 Nature Communications 9, 1–10; Theska et al., 2020 Nature Protocols 15, 2611–2644; Werner et al., 2023 Nature Communications 14, 2095) and relied on the numbers of animals that were actually available (e.g., from crosses or following inbreeding via selection).

Data collection	Data were recorded by hand into notebooks or Microsoft Excel worksheets by Nicholas Levis.
Timing and spatial scale	The species used in this study have a generation time of 4-5 days, which largely determined sampling frequency. In addition animals were sampled as adults, 4-5 days after hatching, because this is when the polyphenism is visible and before the animals suffer from senescence or reduced food supply. For RNA sequencing, mixed stage animals (<1 day to 5 days old) were used to ensure enough input tissue and RNA was available. Biological replicates were at the scale of individual agar plates or liquid culture vials. Initial experiments in P. fissidentatus began February 2021 and the final spr-5 selection experiments ended in June 2023. Sampling timings decisions were made based on previous studies and the rate of development of animals in a given experiment to ensure healthy adults were phenotyped. All experiments were conducted at Indiana University.
Data exclusions	No data were excluded in the study.
Reproducibility	We used replication and statistical tests to ensure reproducibility. We followed sample size conventions of previous studies (e.g., Ragsdale et al., 2013 Cell 155, 922–933; Werner et al., 2017 Scientific Reports 7, 7207; Bui et al., 2018 Nature Communications 9, 1–10; Theska et al., 2020 Nature Protocols 15, 2611–2644; Werner et al., 2023 Nature Communications 14, 2095) and relied on the numbers of animals that were actually available (e.g., from crosses or following inbreeding via selection). For phenotyping, individuals were nested into replicate plates or vials and 30-60 individuals were phenotyped per replicate, with the number of replicates ranging from 3-5 for a given treatment. In the artificial selection portion of the study, all individuals on a plate were phenotyped if there were fewer than 50 adults and this reached as few as seven individuals. No attempts to independently repeat entire experiments were taken.
Randomization	Haphazard picking of individuals placed them into groups for artificial selection. In general, since the species studied were hermaphroditic, divisions were only necessary based on genotype (if mutants) or environmental conditions.
Blinding	During initial phenotyping of spr-5 mutants, Erik Ragsdale was blinded to strain identity and could reliably distinguish mutants from wild-type. For other experiments, blinding was not used because sample preparation and phenotyping were done by the same individual.

Did the study involve field work? ☐ Yes ☒ No

Reporting for specific materials, systems and methods

We require information from authors about some types of materials, experimental systems and methods used in many studies. Here, indicate whether each material, system or method listed is relevant to your study. If you are not sure if a list item applies to your research, read the appropriate section before selecting a response.

Materials & experimental systems

n/a	Involved in the study
☒	☐ Antibodies
☒	☐ Eukaryotic cell lines
☒	☐ Palaeontology and archaeology
☐	☒ Animals and other organisms
☒	☐ Clinical data
☒	☐ Dual use research of concern

Methods

n/a	Involved in the study
☒	☐ ChIP-seq
☒	☐ Flow cytometry
☒	☐ MRI-based neuroimaging

Animals and other research organisms

Policy information about studies involving animals; ARRIVE guidelines recommended for reporting animal research, and Sex and Gender in Research

Laboratory animals	Pristionchus fissidentatus (strains RS5133, EJR1063, EJR1068); Pristionchus pacificus (PS312, EJR1074, EJR1075, RS2651, EJR1085, RS5200B, EJR1088). For phenotyping, animals were 4-5 days old, for RNA sequencing animals were mixed stages ranging from <1 to 5 days old.
Wild animals	No wild animals were used in this study
Reporting on sex	All animals in the study were selfing hermaphrodites.
Field-collected samples	No field collected samples were used in the study.
Ethics oversight	The study did not require an ethical approval.

Note that full information on the approval of the study protocol must also be provided in the manuscript.
